# Improving adherence to multiple medications in older people in primary care: Selecting intervention components to address patient‐reported barriers and facilitators

**DOI:** 10.1111/hex.12595

**Published:** 2017-08-01

**Authors:** Deborah E. Patton, Cathal A. Cadogan, Cristín Ryan, Jill J. Francis, Gerard J. Gormley, Peter Passmore, Ngaire Kerse, Carmel M. Hughes

**Affiliations:** ^1^ School of Pharmacy Queen's University Belfast Belfast UK; ^2^ School of Pharmacy Royal College of Surgeons in Ireland Dublin Ireland; ^3^ School of Health Sciences City University of London London UK; ^4^ Department of General Practice Queen's University Belfast Belfast UK; ^5^ Centre for Public Health Queen's University Belfast Belfast UK; ^6^ School of Population Health University of Auckland Auckland New Zealand

**Keywords:** adherence, behaviour change, community pharmacy, intervention, polypharmacy, qualitative, theoretical domains framework

## Abstract

**Background:**

Medication adherence is vital to ensuring optimal patient outcomes, particularly amongst multimorbid older people prescribed multiple medications. Interventions targeting adherence often lack a theoretical underpinning and this may impact on effectiveness. The theoretical domains framework (TDF) of behaviour can aid intervention development by systematically identifying key determinants of medication adherence.

**Objectives:**

This study aimed to (i) identify determinants (barriers, facilitators) of adherence to multiple medications from older people's perspectives; (ii) identify key domains to target for behaviour change; and (iii) map key domains to intervention components [behaviour change techniques (BCTs)] that could be delivered in an intervention by community pharmacists.

**Method:**

Focus groups were conducted with older people (>65 years) receiving ≥4 medications. Questions explored the 12 domains of the TDF (eg “Knowledge,” “Emotion”). Data were analysed using the framework method and content analysis. Identification of key domains and mapping to intervention components (BCTs) followed established methods.

**Results:**

Seven focus groups were convened (50 participants). A wide range of determinants were identified as barriers (eg forgetfulness, prioritization of medications) and facilitators (eg social support, personalized routines) of adherence to multiple medications. Eight domains were identified as key targets for behaviour change (eg “Social influences,” “Memory, attention and decision processes,” “Motivation and goals”) and mapped to 11 intervention components (BCTs) to include in an intervention [eg “Social support or encouragement (general),” “Self‐monitoring of the behaviour,” “Goal‐setting (behaviour)”].

**Conclusion:**

This study used a theoretical underpinning to identify potential intervention components (BCTs). Future work will incorporate the selected BCTs into an intervention that will undergo feasibility testing in community pharmacies.

## 1 BACKGROUND

Adherence to medications, whereby patients take (or use) their medication in agreement with the recommendations of their clinicians, is vital to ensuring optimal patient outcomes. Adherence is of particular clinical importance in older adults (conventionally those ≥65 years).^1^ This is because older people often suffer from two or more long‐term conditions (ie multimorbidity)^2^ and, therefore, require treatment with multiple medications.^3^, ^4^, ^5^

Non‐adherence to prescribed regimens can result in negative clinical outcomes for older patients, as well as increased use of health‐care resources (eg increased contact with primary health‐care teams, emergency department visits, hospitalizations) and higher associated costs (eg medication wastage).^1^, ^6^ Globally, it is estimated that medication non‐adherence results in annual avoidable costs of approximately US$270 billion.^7^ The scale of the problem is considered to be equivalent to a major disease epidemic and therefore continues to be a key priority for policymakers, researchers and health‐care professionals (HCPs) worldwide.^6^, ^8^ Despite variation in estimated rates of non‐adherence in older adults (range 25%‐75%),^9^ it is clear that there is considerable potential for improvement in this population group.

Adherence is a complex behaviour, and, to date, interventions have shown only limited effectiveness in terms of improving both medication adherence and clinical outcomes.^10^, ^11^, ^12^ For example, a recent update of a Cochrane review involving 182 randomized controlled trials found that a minority of trials, deemed to be at low risk of bias, reported improvements in both medication adherence and clinical outcomes in the intervention groups.^10^ The review authors supported the use of complex interventions when targeting adherence; however, they also highlighted the difficulties surrounding the reproducibility of intervention design and delivery. For example, the specific components of the interventions were often poorly described in published reports, making replication and potential implementation into clinical practice challenging. Another systematic review^13^ that focussed specifically on theory‐based adherence interventions targeting older adults prescribed four or more medications identified a limited number of studies, most of which lacked a robust theoretical underpinning (ie an in‐depth understanding of exactly how the individual components of the intervention will bring about a change in behaviour). The absence of a theoretical base in adherence interventions has been identified as a factor that may be affecting intervention success and effectiveness.^10^, ^13^

To overcome these limitations, this study followed the UK Medical Research Council's (MRC) framework for complex interventions.^14^ As part of initial intervention development work, the MRC recommends that researchers identify existing evidence and establish the intervention's theoretical basis. The aforementioned systematic review of theory‐based adherence interventions delivered to older adults receiving multiple medications^13^ highlighted a lack of published interventions with a robust theoretical underpinning in this area. The study reported here aimed to explore older people's adherence behaviour using the theoretical domains framework (TDF)^15^ as the underpinning model of theoretical determinants of behaviour. The TDF acts as a theoretical lens through which key determinants (ie theoretical domains) of the target behaviour (ie medication adherence) can be identified for targeting with a behaviour change intervention.^16^ Key theoretical domains can then be mapped to appropriate behaviour change techniques (BCTs).^17^, ^18^ The selected BCTs form the “active ingredients” of the intervention and are used to bring about the required changes in the target behaviour. This approach offers a robust, systematic and theory‐based approach to selecting and specifying components of a complex behaviour change intervention.^19^ Therefore, the objectives of this study were to (i) identify determinants (ie barriers and/or facilitators) of adherence to multiple medications from the viewpoint of older adults; (ii) select key TDF domains to target to achieve desired changes; and (iii) map key domains to appropriate BCTs (intervention components) that could be included in an intervention that could feasibly be delivered by community pharmacists.

## 2 METHODS

This study formed part of a multiphase research project that aimed to improve treatment outcomes in older patients by targeting HCPs' clinical behaviours [ie appropriate prescribing and dispensing of polypharmacy (≥4 medications^5^) by general practitioners (GPs) and community pharmacists]^20^, ^21^ and patients' medication adherence behaviours. It was intended at the outset of the project that any intervention to improve adherence to multiple medications in older people in primary care would be delivered by community pharmacists. This was because, in addition to being readily accessible to patients, two recent Cochrane reviews support pharmacists' involvement in interventions to improve patients' use of medications.^10^, ^22^ To explore the behaviour of interest (adherence to multiple medications) in detail, focus groups were conducted with older people who were prescribed four or more regular medications using a TDF‐based topic guide.

Ethical approval was granted by the Office of Research Ethics Committees for Northern Ireland (REC reference 13/NI/0114).

### 2.1 Sampling and recruitment strategy

General practices that had previously participated in a linked study (Cadogan et al.^20^) were approached and asked if they would facilitate patient recruitment into this study. General practices from across the five Health and Social Care Trusts in Northern Ireland were sampled using a purposive sampling approach. Patient recruitment within each practice was overseen by the Northern Ireland Clinical Research Network (NICRN). Inclusion criteria were as follows: patients aged over 65 years, resident in the community, prescribed four or more regular medications and not cognitively impaired. Nurse practitioners from the NICRN screened practice records and issued written invitation letters to patients who met the inclusion criteria. A reply slip was included with the invitation letter. Patients interested in taking part in the study were asked to return the reply slip to a member of the research team (CC) who then made follow‐up contact with patients.

One focus group was scheduled per practice after an adequate number of patients (five patients minimum; 10 patients maximum) confirmed that they could attend. Written informed consent was obtained from all participants before each focus group was convened. Participants were offered an honorarium of £50 for participating in the study.

### 2.2 Focus groups

Focus groups were convened by two members of the research team who acted as moderator (CC) and note‐taker (DP), respectively. Focus groups were held between October 2014 and January 2015, either at the patients' general practice or at another convenient location, (eg local community centre). A semi‐structured topic guide was developed by the research team which included a health psychologist (JF) with expert knowledge of the TDF.^23^ In developing the focus group topic guide, the research team made the decision to use the original 12‐domain version of the framework (TDF1)^15^ rather than the more recent 14‐domain version (TDF2).^24^ This decision was based on the research team's discussion of the importance of the “Nature of the behaviours” domain in the context of older people's adherence behaviour, as previous research has described this behaviour as “routine.”^25^ As the “Nature of the behaviours” domain was thus deemed likely to be important to the target behaviour (ie adherence to multiple medications) and is absent from TDF2,^24^ TDF1^15^ was selected as the theoretical framework for the current study.

Key interview questions (Appendix S1) were developed based on each of the 12 theoretical domains that were included in the original version of the framework (TDF1): “Knowledge,” “Skills,” “Social/professional role and identity,” “Beliefs about capabilities,” “Beliefs about consequences,” “Motivation and goals,” “Memory, attention and decision processes,” “Environmental context and resources,” “Social influences,” “Emotion,” “Behavioural regulation” and “Nature of the behaviours.”^15^ Further descriptions of each theoretical domain are provided in Appendix S2. For example, in relation to the “Motivation and goals” domain, patients were asked *“How important is it to you to take all of your different medicines as the GP has instructed/directed/prescribed?”* Prompts were also included to elicit further information from participants where necessary.

With participants' consent, each focus group was digitally recorded, transcribed verbatim and checked for accuracy. Patient identifiers were removed, and an anonymous code was assigned to each participant.

### 2.3 Data analysis

**Figure 1:**
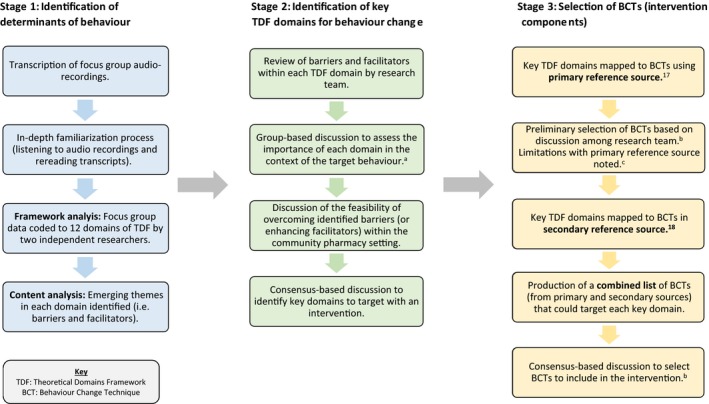
An overview of the three stages involved in data analysis. ^a^A domain was considered to be important if it met the criterion “evidence of verbal agreement or strong beliefs expressed by an individual”. ^b^Selection was based on expected feasibility of BCT delivery in the proposed setting and applicability to target group. ^c^No BCTs were mapped to “Memory, attention and decision processes” and “Social/professional role and identity” domains in the primary reference source

Data analysis comprised three stages: (i) identification of determinants (barriers, facilitators) of adherence, (ii) identification of key TDF domains to target for behaviour change and (iii) mapping of key TDF domains to BCTs (intervention components). Figure 1 provides an overview of these three stages with further details provided in the text below.

#### 2.3.1 Stage 1: Identification of determinants (barriers, facilitators) of adherence

The framework method was used to systematically index and chart data into a framework matrix.^26^ TDF1 was used as the analytical framework whereby each of the 12 domains served as the coding categories.^15^ Following transcription of focus group recordings, an in‐depth familiarization process was undertaken through repeated reading of transcripts, as well as listening to audio recordings. Each transcript was coded independently by at least two members of the research team (DP, CR, CH). Coding was compared and agreed between the coders, and any discrepancies were resolved by discussion. The data were managed using NVivo QSR 10 before being imported into a Microsoft Excel spreadsheet to generate a framework matrix.

Content analysis was then performed inductively on the framework matrix to identify emerging themes relating to barriers and facilitators (ie determinants) of adherence within each TDF domain. A summary of the content analysis was reviewed by three members of the research team (DP, CR, CH), and content themes were agreed upon.

#### 2.3.2 Stage 2: Identification of key TDF domains to target for behaviour change

The second stage of data analysis involved identifying key domains to target with an intervention.^27^ To date, qualitative TDF‐based studies have often involved the use of semi‐structured interviews, and comparatively fewer studies have used focus groups.^28^, ^29^, ^30^, ^31^, ^32^ A study by Bussières et al.^32^ was the only focus group study identified by the research team that described methods used to assess the importance of domains with respect to the target behaviour. This involved the use of frequency counts (ie the number of times that beliefs/statements were mentioned per domain) as one of the assessment criteria.^32^ Although frequency counts have commonly been used as a criterion to assess the importance of domains in TDF‐based interview studies, there are challenges involved in applying this approach to the analysis of focus group data. For example, in a focus group context, there are many verbal and non‐verbal ways that participants can indicate their agreement with other participants (eg short verbal responses, head nodding) which can be difficult to capture accurately from audio recordings.^33^, ^34^ Hence, relying solely on strict frequency counts from the analysed transcripts of the audio recordings may underestimate the importance of a theoretical domain. To overcome this challenge, the research team also took into consideration the expression of strong beliefs (whereby an individual emphasized or re‐iterated a belief) as an indicator of domain importance, in addition to the verbal agreement amongst participants in each group or similarities across focus groups. This adapted criterion (ie “evidence of verbal agreement or strong beliefs expressed by an individual”) guided decisions regarding the importance of each domain to the target behaviour (ie adherence to multiple medications). Key domains to target in an intervention were then selected based on the feasibility of overcoming barriers (or enhancing facilitators) in the proposed setting of community pharmacies and using the wider project's resources. All decisions involved a consensus‐based approach.

#### 2.3.3 Stage 3: Mapping of key TDF domains to BCTs (intervention components)

The process for mapping key theoretical domains to BCTs was guided by methods reported by Cadogan et al.^20^ A mapping table produced by Cane et al.^17^ was used as the primary reference source as it provided the most up‐to‐date guidance on BCT mapping using the current available BCT taxonomy version 1 (BCTTv1).^35^ During group discussions, a number of limitations were noted with the primary reference source.^17^ Firstly, no BCTs had been mapped to “memory, attention and decision processes” or “social/professional role and identity,” and secondly, the mapping process was carried out with TDF2, whereas the current study was based on TDF1. To overcome these limitations, the original mapping matrix developed by Michie et al.^18^ was consulted as a secondary reference source. This matrix^18^ linked 35 BCTs (from a provisional list of BCTs established prior to BCTTv1^35^) to domains in TDF1 as agreed by four experts. A number of BCTs in the two reference sources^17^, ^18^ had overlapping characteristics (eg “information about health consequences” and “information regarding behaviour, outcome”). To avoid potential duplication in the intervention, the research team considered these BCTs to be equivalent and opted to retain the BCT labels reported using BCTTv1.^35^

The BCT selection process was completed by members of the research team and involved a consensus‐based approach. Decisions were informed by the summary of findings from the content analysis of focus group data. In selecting BCTs to target key domains, two main factors were considered (i) the applicability of particular BCTs to the target group (ie older people prescribed multiple medications) and (ii) the expected feasibility of BCT delivery with regard to contextual constraints of the community pharmacy setting.^20^ As part of a linked study, GPs and community pharmacists took part in qualitative interviews (n=30) which explored their views on prescribing and dispensing polypharmacy to older patients, respectively. These study findings, which are reported in a separate publication,^20^ helped to provide contextual information that was relevant to the current intervention (eg time and resource restrictions in current practice). Potential implementation issues (eg likely BCT preparation and delivery time) were taken into consideration at this early stage of intervention development to help exclude BCTs that were unlikely to be feasible for delivery in primary care by community pharmacists.

## 3 RESULTS

### 3.1 Participant characteristics

Seven of the ten general practices that participated in the previous linked study^20^ agreed to facilitate patient recruitment, and seven focus groups were convened (one per practice). Overall, 50 participants (60% female) were recruited, with each focus group comprising between five and 10 participants and ranging in duration from 65‐123 minutes (Table 1). Data saturation was reached by the seventh focus group as no new themes were emerging at this point.

**Table 1:** Characteristics of focus groups

Focus group number	Number of participants	Male:female ratio	Duration (minutes)	Health and social care trust area (urban/rural)
1	10	3:7	102	1 (urban)
2	9	5:4	123	2 (urban)
3	7	2:5	88	3 (rural)
4	6	2:4	87	4 (rural)
5	6	2:4	65	2 (urban)
6	7	3:4	84	4 (urban)
7	5	3:2	69	3 (rural)

### 3.2 Summary of key findings from Stage 1 (identification of determinants of adherence)

A wide range of reported barriers and facilitators of adherence to multiple medications were identified within each theoretical domain; these are presented in Table 2 together with illustrative quotes.

**Table 2:** Determinants (ie barriers and/or facilitators) of older patients' adherence behaviour identified within each TDF domain and illustrative quotes

TDF domain	Determinants (ie barriers and/or facilitators) of adherence to multiple medications	Illustrative quotes
Knowledge	Lack of/incorrect knowledge of clinical indication, treatment duration or administration timing (barrier) Lack of/incorrect knowledge of the consequences of adherence or non‐adherence (barrier) Extent of knowledge on medication side‐effects (barrier or facilitator)^a^	“I wasn't aware, and I'll have to read the boxes again. I wasn't aware of, of the time of the day or night…” (FG07PT02) “You know, a build‐up of this, that and the other, you just sort of wonder can that be good or would you be better off taking a break…” (FG05PT02) “Sometimes the less you know the better, just take it.” (FG05PT01)
Beliefs about consequences	Concerns about medication side‐effects/long‐term consequences of adherence or non‐adherence (barrier) Belief that missed doses cause no harm (barrier) Belief that medication is unnecessary and/or lacks benefit (barrier) Belief that non‐adherence has negative outcomes (eg hospitalization, mortality) (facilitator) Belief that medication is necessary and/or beneficial (eg improves quality of life, prolongs survival) (facilitator) Return of symptoms (facilitator)	“Well, blood pressure is very serious, I would take my blood pressure tablet every day. I'm on aspirin, I take that every day. See this is why I laughed when I got the letter and it said, you know, ‘Four tablets plus'. I am officially down as four tablets plus but I don't take four tablets plus…” (FG03PT06) “I remember at one stage thinking I don't think them tablets are doing me any good, I would say to the wife, ‘You wouldn't be taking them no more'. I said, ‘I want to stop, I don't think they're doing me a lot of good…'” (FG02PT04) “…I've never stopped taking them but I sort of wondered if I stopped taking these what would happen but I tried it for a wee while but my blood pressure went away up. And then it takes a wee while for the tablets to be effective again.” (FG04PT01)
Emotion	Anxiety about side‐effects/long‐term consequences of adherence or non‐adherence (barrier) Anxiety about potential consequences of non‐adherence (facilitator)	“Well, I would worry about the side effects but I know I have no choice but take them.” (FG01PT04) “I'd be afraid of not taking them, I don't know what the effect would be but I'd be afraid if I didn't take them that it would affect me badly.” (FG07PT02)
Skills	Lack of physical skills to take medications as prescribed (eg ability to swallow medications, poor manual dexterity) (barrier)	“But I couldn't, I couldn't actually physically get them out, [Out of the thing, yeah] trying to get the back open.” (FG01PT09) “Sometimes it's quite difficult to, to pop them out of the foil.” (FG06PT02)
Beliefs about capabilities	Belief about lack of physical capability (see “Skills” domain) (barrier) Belief that medication use is not difficult (facilitator)	“And I would say, ‘Excuse me, I can't take those, [Can't swallow] no, can you give me those ones that's in the water?'…” (FG01PT01) “But it's the top, if you've one of those tops they're impossible if you've arthritic hands.” (FG01PT06) “I've no difficulty there with anything…there, as long as I'm able to take the tablets that's the main thing…” (FG04PT05)
Environmental context and resources	Access to medications (eg at weekends) (barrier) Changing environment (eg on holidays, day trips) (barrier) Physical resources (eg MDS, medication lists) (facilitator)	“…you have to make sure you have everything with you and sometimes you'd be in meetings or something like here and the time you're supposed to take it is gone by.” (FG03PT03) “I get it in a bubble pack [MDS] for the week and he leaves it out morning and evening, it's just so easy in case you forget them…” (FG05PT04)
Motivations and goals	Goals to reduce the total number of prescribed medications (barrier) Relative priority placed on medications that patients deemed to be of greater importance (barrier/ facilitator)^b^ High intrinsic motivation to take medications as prescribed (facilitator) Goals to avoid hospital admission, maintain driving licence, clinical goals (eg symptom control) (facilitator)	“You decide what's the serious ones and if you run out of a lesser tablet, well it's not as dangerous, you can wait till you get to the pharmacist, you know. There's a couple of my tablets that, well I need to take them but they're not as important if you know what I mean as the blood pressure tablets…” (FG03PT03) “Well I think it's very important for me too because I would have…kidney failure or kidney disease and I think if I didn't do me things right I might end up in hospital again where I don't want to be.” (FG07PT03)
Behavioural regulation	Systems that alert patients to missed doses (eg MDS) (facilitator) Practical and reminder strategies (eg placement of medication in a visually prominent place) (facilitator) Action planning (eg planning administration times) (facilitator) Self‐monitoring of medication use and outcomes (eg blood glucose, symptom control) (facilitator)	“And I put it [MDS] down beside the kettle because I know I'm going to the kettle in the mornings, the tablets are there for me.” (FG07PT03) “I have a wee weekly box and I take so many tablets in the morning they're divided between two compartments but I do all my week's drugs on a Sunday night so they're all done.” (FG01PT09)
Memory, attention and decision processes	Forgetting to take medications as prescribed (barrier) Paying attention to medications deemed to be of higher importance (barrier/facilitator)^b^ Paying attention to medications when out of normal context (eg on holidays, at meetings) (facilitator) Making decisions regarding medication use without consulting a HCP (eg reducing doses, non‐persistence) (barrier) Involving HCPs in decisions regarding medication use (facilitator)	“So obviously I've forgotten, not that I'm that fond of statins anyway because they keep giving me pains, they're desperate.” (FG06PT07) “I have at several times…with different medications cut down to see how I can go, I've never actually stopped…that I cut it out altogether, no I haven't done that.” (FG04PT02) “Sometimes when I go on holiday I don't take my fluid one. I just‐ but it's combined with my blood pressure tablet…so I'm cutting both of them out but I do, for a few days anyway.” (FG01PT06)
Social influences	Social support/pressure from family (facilitator) Social support/pressure from HCPs (facilitator) Lack of (or withdrawal of) social support from family (eg death of spouse) (barrier)	“‘You're not taking your tablet, I know by the look on your face'… that sort of reacts to you because the girl [Diabetic nurse] knows you and you know the girl, it's not as if she's a stranger.” (FG01PT01) “My wife passed away last Christmas and I, I find it difficult to manage [my] tablets. She remembered every time I had to take a tablet and sometimes I was going days without certain tablets…” (FG01PT10)
Social/ professional role and identity	Patient autonomy (ie viewing medication use as their own responsibility) (facilitator)	“Everyone would be responsible for themselves.” (FG04PT06) “…I'm the one that's been affected by it so… as far as I'm concerned… it's my responsibility to do it.” (FG06PT01)
Nature of the behaviours	Having a personalized routine (eg linked to meal times) (facilitator) Lack of routine or ineffective routine (barrier) Return of symptoms (direct experience) (facilitator)	“Well, I used to worry about, as I say, taking the tablets and so I developed a wee routine, you know. Here's me, I'll take them this way. So I take the, the wee one in the morning and then start eating my porridge…” (FG02PT06) “It's no difficulty for me because as soon as I have my breakfast into the kitchen, into the cupboard, get them out, that's it. It's just routine.” (FG01PT08)

HCP, Health‐care professional; MDS, Monitored Dosage System.

a This determinant could be a barrier or facilitator depending on the individual circumstances.

b This determinant facilitates adherence to the medication the patient deems to be of greater importance but acts as a barrier to medications deemed to be less important.

### 3.3 Summary of key findings from Stage 2 (Identification of key domains)

Based on the research team's review of the summary findings from Stage 1, all 12 domains were considered to be important in respect to the target behaviour (adherence to multiple medications). Through group consensus, eight of the 12 domains were selected as key domains to target as part of a community pharmacy‐based intervention: “Knowledge,” “Beliefs about consequences,” “Motivation and goals,” “Environmental context and resources,” “Social influences,” “Memory, attention and decision processes,” “Behavioural regulation” and “Nature of the behaviours”. Four domains were not selected as key target domains: “Social/professional role and identity,” “Beliefs about capabilities,” “Skills” and “Emotion.”

### 3.4 Summary of key findings from Stage 3 (Mapping of key domains to BCTs)

Forty‐one BCTs were identified from the two reference sources^17^, ^18^ and considered for inclusion in the intervention. Eleven BCTs were subsequently selected for inclusion in an intervention [“Information about health consequences,” “Feedback on behaviour,” “Goal‐setting (outcome),” “Review of outcome goal,” “Goal‐setting (behaviour),” “Review of behaviour goal,” “Action planning,” “Prompts and cues,” “Restructuring the physical environment,” “Social support or encouragement (general)” and “Self‐monitoring of the behaviour”]. Table 3 presents the selected 11 BCTs mapped to key TDF domains (Appendix S3 includes further details of BCTs that were not selected for inclusion in the intervention).

**Table 3:** Final selection of BCTs to target each key domain and include as components of an intervention to improve adherence to multiple medications in older people

Key TDF domain	Behaviour Change Techniques(BCTs) selected to target the TDF domain
Knowledge	Information about health consequences^a^ /Information regarding behaviour, outcome^b^
	Feedback on behaviour^a^
Beliefs about consequences	Self‐monitoring^a^
	Information regarding behaviour/outcome^b^
	Feedback^b^
Environmental context and resources	Restructuring the physical environment^a^/ Environmental changes^b^
	Prompts and cues^a^
Motivation and goals	Goal‐setting (outcome)^a^ Goal‐setting (behaviour)^a^ Review of outcome goal^a^ Review of behaviour goal^a^ Goal/target specified: behaviour or outcome^b^
	Action planning^a^
	Social processes of encouragement, pressure, support^b^
	Information regarding behaviour, outcome^b^
Behavioural regulation	Self‐monitoring (of behaviour)^a^
	Goal/target specified: behaviour or outcome^b^
	Planning, implementation^b^
	Prompt/triggers/ cues^b^
Memory, attention and decision processes	Self‐monitoring^b^
	Planning, implementation^b^
	Prompts/trigger/cues^b^
Social influences	Social support or encouragement (general)^a^/ Social processes of encouragement, pressure, support^b^
Nature of the behaviours	None identified^c^

a Identified from primary reference source.^17^

b Identified from secondary reference source.^18^

c This domain was not included in either reference source; therefore, no BCTs were mapped to this domain. This domain will be targeted indirectly using the selected BCTs that were mapped to the other key domains (eg BCT: prompts and cues that mapped to environmental context and resources).

## 4 DISCUSSION

This article reports findings from the systematic process that was used to identify key theoretical determinants of adherence to multiple medications in older people and select intervention components (BCTs) to include in an intervention for delivery by community pharmacists. The focus group findings highlight the wide range of barriers and facilitators perceived to be influencing older patients' adherence behaviour, and the subsequent challenge for researchers in selecting both key domains to target and intervention components (BCTs) to bring about behaviour change.^20^

Four domains were not selected because they did not contain barriers/ facilitators that were considered feasible to target within the available project resources and selected setting of community pharmacy. For example, under the “Skills” domain, patients discussed the physical skills involved in opening mediation packaging and swallowing oral dosage formulations. These are recognized issues that are important to consider in ensuring appropriate use of multiple medications in older people.^36^ However, it was beyond the scope of the current project to improve patients' physical skills relating to manual dexterity or swallowing ability. Hence, the “Skills” domain was not considered for intervention targeting. Instead, it was intended that the barriers identified under this domain would be addressed indirectly by ensuring that appropriate types of formulations and medication packaging were issued to patients (“Environmental context and resources”).

This study contributes to the growing body of literature on the application of the TDF in designing behaviour change interventions. While the TDF was originally developed to investigate the implementation of evidence‐based practices by HCPs,^15^ it is now being used to explore patient behaviours.^37^, ^38^ This study highlights the usability of the TDF as the underpinning theoretical model in focus group studies examining patient behaviours. Incorporating a theory base into the development of this intervention will allow explicit links to be made between intervention components and outcomes and ultimately help to understand the causal mechanisms underlying the intervention's effects.^39^

The importance of routine to patients' adherence behaviour is consistent with previous qualitative studies^25^, ^40^, ^41^ which, in contrast to the current study, were not underpinned by a theoretical framework of behaviour change. As “routine” was coded under the “Nature of the behaviours” domain, which has since been removed from the framework in TDF2,^24^ the focus group findings support our rationale for the selection of TDF1^15^ as the underpinning model for the current project. Based on the focus group findings, it was evident that the “Nature of the behaviours” domain would need to be targeted, albeit indirectly, in an intervention to improve adherence to multiple medications in older people. It is proposed that BCTs selected to target other key domains will influence the routine nature of patients' adherence behaviours (eg “prompts/cues,” “self‐monitoring of the behaviour”) and, hence, target the “Nature of behaviours” domain indirectly.^42^

This study represents a further advancement of the application of the TDF^15^, ^24^ and BCT taxonomy (v1)^35^ in the development of patient‐targeted behaviour change interventions. A recent study by McCullough et al.^38^ mapped key TDF domains to BCTs using the original mapping protocol developed by Michie et al.^18^ In the current study, the work by Cane et al.^17^ served as the primary mapping reference source and provided the most recent guidance in completing the BCT mapping process.

As previously outlined, the project modelled the MRC framework^14^ in that it endeavoured to incorporate both an evidence base and a theory base into the intervention development phase. In operationalizing the MRC framework, we also considered practical implementation issues during initial BCT selection. Failure to consider these practicalities at an early stage can result in “*weaker interventions that are harder to evaluate, less likely to be implemented and less likely to be worth implementing.”* 14 Throughout the decision‐making process regarding BCT selection, the research team was conscious of the time constraints in primary care which have been well publicized.^43^, ^44^, ^45^ Drawing on our previous experience of developing an intervention targeting HCPs,^20^ as well as knowledge of relevant literature that multifaceted interventions are not necessarily more effective in changing target behaviours than single‐component interventions,^46^ we aimed to keep the intervention as simple as possible. It was clear that inclusion of all 41 identified BCTs would make the intervention too complex and impossible to implement in the proposed setting. Hence, it was beneficial if BCTs targeted multiple key domains and this was taken into consideration during the BCT selection process. For example, the BCT “Action planning” was selected instead of the BCT “Graded tasks, starting with easy tasks,” as the former targets three key domains (“Motivation and goals,” “Behavioural regulation” and “Memory, attention and decision processes”), whereas the latter targets only one key domain (“Motivation and goals”).

The BCT “Threats” was considered to be an inappropriate method for attempting to change older patients' “Beliefs about consequences” of non‐adherence. This is because threats can evoke negative emotions which could be detrimental to the patient‐HCP relationship, and this does not align with the person‐centred approach to medicines optimization that is advocated by organizations such as the UK National Institute for Health and Care Excellence (NICE).^47^ Conversely, the BCT “Information about health consequences” was considered more appropriate for the target intervention recipients (see Appendix S3 for further explanations for selection or non‐selection of BCTs).

The “Nature of the behaviours” domain was the only domain that did not map directly to any BCTs in either reference source.^17^, ^18^ This is because this domain is considered to be distinct from the other domains, in that it represents the “essential characteristics of the behaviour” (dependent variable), rather than a predictor of the behaviour (independent variable).^24^ However, as discussed previously, it will be targeted indirectly with BCTs (eg “Prompts/cues”) selected to target other key TDF domains.^42^

As the methodology of TDF‐based intervention development continues to evolve, guidelines on the “best practice” approach for selecting BCTs to include in an intervention would serve as a valuable resource to those working in this field of research.

### 4.1 Strengths and limitations

The details reported in this article follow guidelines^48^ produced by a Workgroup for Intervention Development and Evaluation Research (WIDER) which recommend reporting on: *“1) The intervention development; 2) The change techniques used in the intervention; 3) The causal processes targeted by these change techniques.”* By making explicit links between the intervention components and key determinants of behaviour change, this will facilitate our understanding of how the intervention exerts its effect. Specifying the intervention content in terms of BCTs from an established taxonomy (BCTTv1)^35^ will facilitate replication and implementation of the intervention in future evaluations and, ultimately, into clinical practice, if the intervention is shown to be effective. The study also followed previous recommendations on the effective application of the TDF.^19^

Due to the extensive reporting requirements that are now recommended to ensure that interventions can be replicated, we are unable to provide detailed descriptions in the current article of how BCTs will be delivered in an intervention.^48^, ^49^ A future paper will describe how the 11 selected BCTs were operationalized in the design of an intervention so that it could be delivered to older people in the community pharmacy setting. The results from this study have provided important contextual information that will inform exactly how each BCT will be operationalized as part of a complex intervention. The selection of intervention components in this study has focused on the patient's perspective to taking several medications as prescribed, however, as part of the future testing of the intervention the views and opinions of those involved in intervention delivery (ie community pharmacists) will be sought to inform the need for any refinements.

As a limitation of the study, it must be noted that focus group participants were self‐selected and their level of adherence was not formally measured. Nonetheless, variation in the sample of participants was evident, ranging from those who reported no issues with adherence to those who reported frequent non‐adherence behaviours. The inclusion of both adherent and non‐adherent patients enabled the exploration of both facilitators and barriers to the target behaviour.

It must also be noted that the intervention development work is underpinned by a qualitative study. Although this allowed an in‐depth exploration of the target behaviour, the findings are not readily generalizable to the wider population of older people in primary care. However, the sampling strategy incorporated participants from both urban and rural areas from across the regions of Northern Ireland which helps to increase the transferability of the focus group findings.

## 5 CONCLUSIONS

Identifying key domains from focus group data and mapping to BCTs has provided the basis for designing an intervention to improve adherence to multiple medications. Future work will focus on incorporating the selected BCTs into an intervention for community pharmacists to deliver to this group of patients. The intervention will undergo feasibility testing at a later stage of the project before any larger‐scale trial evaluation is conducted.

## AUTHORS' CONTRIBUTIONS

Deborah E. Patton (joint first author) contributed to the development of the focus group topic guide, took notes at focus groups, analysed the data and led the writing of the article. Cathal A. Cadogan (joint first author) led on the development of the focus group topic guides, facilitated the focus groups, contributed to review of results and the writing of the article. Cristín Ryan codeveloped the research programme, contributed to the development of the focus group topic guide, analysed the data and contributed to review of results and writing of the article. Jill J. Francis contributed to the design of the study, provided health psychology expertise, critiqued the focus group topic guide and contributed to review of results and writing of the article. Gerard J. Gormley contributed to the design of the study and critiqued the focus group topic guide. Peter Passmore contributed to the design of the study. Ngaire Kerse contributed to the design of the study. Carmel M. Hughes (PI) codeveloped the research programme, contributed to the development of the focus group topic guide, analysed the data and contributed to review of results and writing of the article. All authors commented on drafts and approved the final manuscript.

## CONFLICT OF INTEREST

The authors declare that they have no competing interests.

## CONSENT FOR PUBLICATION

Not applicable.

## ETHICAL APPROVAL AND CONSENT TO PARTICIPATE

Ethical approval was granted by the Office of Research Ethics Committees for Northern Ireland (REC reference 13/NI/0114). Written informed consent was obtained from all participants before each focus group was convened.
