# Supplementary material for: Improving adherence to multiple medications in older people in primary care: Selecting intervention components to address patient‐reported barriers and facilitators
Source: Health Expect. 2017 Aug 1;21(1):138–48. doi: 10.1111/hex.12595 (PMC5750691; doi:10.1111/hex.12595)
Supplement: Supplementary file 1 [file HEX-21-138-s001.docx]

# Appendix S1: TDF questions from the focus group topic guide

I’d like you all to think for a moment about all the different medicines that you take every day. Now, if you look at the coloured sheet in front of you, you will see three different statement. I would like you to think about which of these three answers best applies to you. **[*Knowledge*]**

- ‘I know what **every medicine** that I take is for’
- ‘There is **one medicine** that I take and I don’t know what it is for’
- ‘There are **several medicines** that I take and I don’t know what that they are for’

Please remember that whatever answer applies to you is ok. So, raise your hand if you don’t know what… several of the medicines that you take are for? ...one of the medicines that you take is for? ...every medicine that you take is for?

And, then if you look at the other coloured sheet in front of you, I would like you to think about which of these three statements best applies to you **[*Skills*]**

- ‘I know how to take **all of my medicines**’
- ‘I don’t know how to take **one of my medicines**’
- ‘I don’t know how to take **several of my medicines**’

As I said before, whatever answer applies to you is ok. So, raise your hand if you… don’t how to take several of your medicines? ... don’t how to take one of your medicines?... know how to take all of your medicines?

And what about making sure that you take your medicines as advised? Whose role do you think it is it to make sure that this happens?

As I mentioned at the start, we asked you to come here today because you all take several medicines every day.

How important is it to you to take all of your different medicines as the GP has instructed/directed/prescribed? [***Motivation and goals***]

When would it be less important to you to take all of your different medicines as the GP has instructed/directed/prescribed? [***Motivation and goals***]

What do you think the benefits are of taking all of your medicines as prescribed? **[*Beliefs about consequences*]**

What do you think the disadvantages are to taking all your medicines as prescribed? **[*Beliefs about consequences*]**

Are the benefits of taking all of your medicines worth the possible disadvantages? **[*Beliefs about consequences*]**

If you were think about how you fit having to take several medicines into your daily routine when you’re at home:

- What makes this difficult to do when you are at home? **[*Environmental context and resources*]**
- What helps you to do this when you are at home? **[*Environmental context and resources*]**

Do you feel confident that you can take all your medicines as advised by your GP or community pharmacist? **[*Beliefs about capabilities*]**

What are the biggest problems/challenges in having to take at least four medicines every day? **[*Beliefs about capabilities*]**

What would help you to overcome these problems/challenges? ***[Behavioural regulation]***

**Prompt:** Practical strategies/procedures

How would you feel if you didn’t take your medicines as advised? ***[Emotion]***

Does anyone have any routines or habits that help them to make sure they take their medicines as instructed/directed/prescribed? ***[Nature of the behaviours]***

And apart from forgetting to take your medicines, would you ever decide not to take your medicines as instructed/directed? What would be the reasons that you would deliberately decide not to take the medicines as instructed/directed? **[Memory, attention and decision processes]**

**Prompt:** Any other reasons?

Who else influences your decisions about whether you to take your medicines as instructed/directed? ***[Social influences]***

**Prompt**: *What about the influence of family? carers?*

# Appendix S2: Domains in TDF1^(19)^ and descriptions (adapted from Francis et al^(50)^)

| **Domain label** | **Descriptions** |
| --- | --- |
| **Knowledge** | - Knowledge related to medications, regimens and clinical conditions |
| **Skills** | - Abilities (i.e. physical skills) required to take several medications as prescribed |
| **Social/professional role and identity** | - Whether patients see the behaviour as their own responsibility or as someone else’s |
| **Beliefs about capabilities** | - How confident the patient feels about being able to adhere to multiple medication regimens (i.e. ease or difficulty of the behaviour) |
| **Beliefs about consequences** | - Perceptions about the outcomes of taking (or not taking) several medications as prescribed (benefits and unwanted side effects) |
| **Motivation and goals** | - Strength of intention to adhere to the medication regimen - Priorities, perceived importance and commitment to taking several medications as prescribed |
| **Memory, attention and decision processes** | - Decision-making – whether patients choose to take certain medicines over others; whether they decide to have occasional breaks from taking medications - Memory (i.e. whether adherence to polypharmacy is problematic because patients simply forget) |
| **Environmental context/resources** | - Physical factors (e.g. difficulty swallowing large tablets) - Financial and healthcare system factors (e.g. difficulty accessing a repeat prescription service) - Circumstances (e.g. location, time) under which the behaviour is occurring (e.g. whether adherence is more difficult when patient is away from home) |
| **Social influences** | - External pressure/influence from others (e.g. healthcare professionals, carers, family, other patients) - Whether patients take several medications as prescribed simply because they are told to |
| **Emotion** | - Affect (positive or negative) - Feelings towards taking (or not taking) several medications as prescribed |
| **Behavioural regulation** | - Whether patients have ways of monitoring their medication adherence/non-adherence and outcomes - Whether patients have difficulty translating intentions to take several medications as prescribed into action |
| **Nature of the behaviours** | - Past experiences of adherence/non-adherence to polypharmacy - Whether medication adherence is a routine/automatic behaviour for the patient |

# Appendix S3: Table outlining the BCTs that mapped to each key TDF domain and rationale for selection of BCTs to include in an intervention to improve adherence to multiple medications in older people

| **Key TDF domain** | **BCTs identified to target key domain** | **BCT selected**  ✓ = YES  X = NO | **Rationale for selecting BCT(s) to include in the intervention** |
| --- | --- | --- | --- |
| Knowledge | - Information about health consequences^a^ /Information regarding behaviour, outcome^b^ | ✓ | Verbal information could be provided by outlining positive consequences of adhering to medications and negative consequences of non-adherence. This BCT also mapped to ‘Beliefs about consequences’ and ‘Motivation and goals’ domains—see below. *Note: These BCTs were considered to be equivalent.* |
|  | - Feedback on behaviour^a^ | ✓ | Patients could be provided with verbal feedback on their adherence behaviours. This BCT also mapped to ‘Beliefs about consequences’ domain—see below. |
|  | - Antecedents (e.g., distraction, adding objects)^a^ | X | Due the restricted time environment in the proposed setting of primary care, BCTs in this grouping were not deemed to be necessary in addition to previously selected BCTs–see above. |
|  | - Biofeedback^a^ | X | Providing feedback on the outcomes of behaviour (e.g., improvements in clinical parameters) would be difficult to achieve in the context of community pharmacy and with the current intervention which targets a heterogeneous patient group. |
| Beliefs about consequences  Beliefs about consequences  (cont’d) | - Comparative imagining of future outcomes^a^ - Pros and cons^a^ - Covert conditioning^a^ | X | Due the restricted time environment in the proposed setting of primary care, these BCTs were not deemed to be necessary in addition to BCTs previously selected to target the ‘Knowledge’ domain (‘Feedback on behaviour’, ‘Information about health consequences’) which also mapped to ‘Beliefs about consequences’ domain—see below. |
|  | - Emotional consequences^a^ | X | It is beyond the scope of the current study to target patients’ emotions directly and this BCT would likely require extensive delivery over a long period of time. |
|  | - Salience of consequences^a^ | X | This BCT would likely require significant and detailed preparation which would be difficult to achieve in the context of the current intervention which targets a heterogeneous patient group. |
|  | - Covert sensitization^a^ - Anticipated regret^a^ | X | These BCTs were not considered to be appropriate for inclusion, as the current intervention will focus on improving medication adherence (i.e. a wanted behaviour) rather than an unwanted behaviour. |
|  | - Social and environmental consequences^a^ | X | This BCT is not applicable to the target audience as participants in the focus groups focused more on the health consequences of the target behaviour. |
|  | - Vicarious reinforcement^a^ | X | This BCT is not applicable to the target audience as the behaviour is highly individualised based on the patients’ own medications and medical conditions. |
|  | - Threat^a^ | X | Threats were not considered to be appropriate for attempting to change patient’s adherence behaviour as they could evoke negative emotions and harm the patient-HCP relationship. |
|  | - Self-monitoring^a^ | ✓ | Patients could self-monitor their medication use via a personalised daily medication diary. This BCT also mapped to ‘Behavioural regulation’ and ‘Memory, attention and decision processes’ domains–see below. |
|  | - Persuasive communication^b^ | X | This BCT would likely require delivery over an extended time period which was not seen as feasible in the proposed setting of community pharmacies. |
|  | - Information regarding behaviour/outcome^b^ | ✓ | See ‘Knowledge’ domain above. |
|  | - Feedback^b^ | ✓ | Equivalent to ‘Feedback on behaviour’. See ‘Knowledge’ domain above. |
| Environmental context and resources | - Restructuring the physical environment^a^/ Environmental changes^b^ | ✓ | Changes to the physical environment, such as non-child resistant bottles, re-packaging medications in MDS, could be made to facilitate adherence. *Note: These BCTs were considered to be equivalent.* |
|  | - Discriminative (learned) cue^a^ | X | It is beyond the scope of this project to offer rewards or incentives. |
|  | - Prompts and cues^a^ | ✓ | Patients could use reminder stickers or strategic placement of medications in a visually prominent place to prompt medication use (e.g., kitchen bench). This BCT also mapped to ‘Behavioural regulation’ and ‘Memory, attention and decision processes’ domains—see below. |
|  | - Restructuring the social environment^a^ | X | It is beyond the scope of this project to restructure the social environment. |
|  | - Avoidance/changing exposure to cues for the behaviour^a^ | X | This BCT was not considered to be appropriate for inclusion, as the current intervention will focus on improving medication adherence (i.e. a wanted behaviour) rather than an unwanted behaviour. |
| Motivation and goals | - Goal setting (outcome)^a^ - Goal setting (behaviour)^a^ - Review of outcome goal^a^ - Review of behaviour goal^a^ - Goal/target specified: behaviour or outcome^b^ | ✓ | Patients could be prompted to set goals related to positive outcomes of medication use (e.g., fewer symptoms, avoiding hospital admissions) and/or medicine use (i.e. behaviour) goals tailored to their own medications. These goals could be reviewed at follow-up appointments. *Note: ‘Goal/target specified: behaviour or outcome’ encompasses the following BCTs: Goal setting (outcome), Goal setting (behaviour), Review of outcome goal and Review of behaviour goal.* This BCT also mapped to ‘Behavioural regulation’ domain—see below. |
|  | - Action planning^a^ | ✓ | Patients could make explicit plans with their HCPs as to how to use their medication as prescribed. This BCT also mapped to ‘Memory, attention and decision processes’ and ‘Behavioural regulation’ domains— see below. |
|  | - Contract^b^ - Graded tasks, starting with easy tasks^b^ | X | Due to the limited available time in the proposed setting of primary care, these BCTs were not deemed to be necessary in addition to previously selected BCTs (e.g., goal setting, action planning). |
|  | - Rewards; incentives^b^ | X | It is beyond the scope of this project to offer rewards or incentives. |
|  | - Increasing skills: problem solving, decision making, goal setting^b^ | X | This BCT encapsulates some aspects of previously selected BCTs (e.g., ‘Goal setting (behaviour)’). |
|  | - Social processes of encouragement, pressure, support^b^ | ✓ | Patients could be advised to seek additional support from family or friends or HCPs could encourage adherence. This BCT also mapped to ‘Social influences’ domain—see below. |
|  | - Persuasive communication^b^ - Motivational interviewing^b^ | X | These BCTs would likely require delivery over an extended time period which was not seen as feasible in the proposed setting of primary care. |
|  | - Information regarding behaviour, outcome^b^ | ✓ | Refer to ‘Knowledge’ domain above. |
| Behavioural regulation | - Self-monitoring (of behaviour)^a^ | ✓ | Equivalent to ‘Self-monitoring’—refer to ‘Beliefs about consequences’ domain above |
|  | - Goal/target specified: behaviour or outcome^b^ | ✓ | Refer to ‘Motivation and goals’ domain above. |
|  | - Contract^b^ - Use of imagery^b^ | X | Due to the restricted time environment in the proposed setting of primary care, these BCTs were not deemed to be necessary in addition to previously selected BCTs. |
|  | - Planning, implementation^b^ | ✓ | Equivalent to ‘Action planning’. Refer to ‘Motivation and goals’ domain above. |
|  | - Prompt/triggers/ cues^b^ | ✓ | Equivalent to ‘Prompts and cues’. Refer to ‘Environmental context and resources’ domain above. |
| Memory, attention and decision processes | - Self-monitoring^b^ | ✓ | Refer to ‘Beliefs about consequences’ domain above. |
|  | - Planning, implementation^b^ | ✓ | Equivalent to ‘Action planning’. Refer to ‘Motivation and goals’ domain above. |
|  | - Prompts/trigger/cues^b^ | ✓ | Equivalent to ‘Prompts and cues’. Refer to ‘Environmental context and resources’ domain above. |
| Social influences | - Social support or encouragement (general)^a^/ Social processes of encouragement, pressure, support^b^ | ✓ | Refer to ‘Motivation and goals’ domain above. *Note: These BCTs were considered to be equivalent.* |
|  | - Social comparison^a^ - Vicarious reinforcement^a^ - Identification of self as role model^a^ | X | These BCTs are not applicable to the target audience as the behaviour is highly individualised based on the patients’ own medications and medical conditions. |
|  | - Information about others approval^a^ - Social reward^a^ | X | Due the restricted time environment in the proposed setting of primary care, these BCTs were not deemed to be necessary in addition to the previously selected BCT—see above. |
|  | - Social support (emotional)^a^ - Social support (practical)^a^ | X | These BCTs encapsulate aspects that are already covered by the previously selected BCT ‘Social support or encouragement (general)’. |
|  | - Restructuring the social environment^a^ | X | It is beyond the scope of this project to restructure the social environment. |
|  | - Modelling or demonstrating the behaviour^b^ | X | This BCT would likely require significant and detailed preparation which would be difficult to achieve in the context of the current intervention which targets a heterogeneous patient group. |
| Nature of the behaviours | None identified | N/A | This domain was not included in either reference source, therefore, no BCTs were mapped to this domain. This domain will be targeted indirectly using the selected BCTs that were mapped to the other key domains (e.g., BCT: prompts and cues that mapped to environmental context and resources). |
| ^a^=Identified from primary reference source^17^  ^b^=Identified from secondary reference source^18^ | | | |
